# Preventing Persistence of HPV Infection with Natural Molecules

**DOI:** 10.3390/pathogens12030416

**Published:** 2023-03-06

**Authors:** Antonio Simone Laganà, Vito Chiantera, Sandro Gerli, Sara Proietti, Elisa Lepore, Vittorio Unfer, Jose Carugno, Alessandro Favilli

**Affiliations:** 1 Unit of Gynecologic Oncology, ARNAS “Civico-Di Cristina-Benfratelli”, Department of Health Promotion, Mother and Child Care, Internal Medicine, and Medical Specialties (PROMISE), University of Palermo, 90127 Palermo, Italy; 2 Section of Obstetrics and Gynecology, Department of Medicine and Surgery, University of Perugia, 06123 Perugia, Italy; 3 Center for Research in Perinatal and Reproductive Medicine, University of Perugia, 06123 Perugia, Italy; 4 R&D Department, Lo.Li. Pharma, 00156 Rome, Italy; 5 AGUNCO Obstetrics & Gynecology Center, Saint Camillus International University of Health Sciences (UniCamillus), 00131 Rome, Italy; 6 Minimally Invasive Gynecology Unit, Obstetrics, Gynecology and Reproductive Sciences Department, Miller School of Medicine, University of Miami, Miami, FL 33136, USA

**Keywords:** EGCG, folic acid, vitamin B12, HA, HPV infection, persistence

## Abstract

Human papillomavirus (HPV) infection is one the most common sexually transmitted infections worldwide. In most cases, the infection is temporary and asymptomatic; however, when persistent, it may lead to lesions that can evolve into cancer in both women and men. Nowadays, prophylactic vaccination is the primary preventive strategy for HPV infections, but vaccines do not cover all types of HPV strains. Scientific research has uncovered the beneficial role of some natural supplements in preventing persistent HPV infections or treating HPV-related lesions. We review the current insight into the roles of natural molecules in HPV infection with a special focus on epigallocatechin gallate (EGCG), folic acid, vitamin B12, and hyaluronic acid (HA). Specifically, EGCG from green tea extracts plays a critical role in suppressing HPV oncogenes and oncoproteins (E6/E7), which are responsible for HPV oncogenic activity and cancer development. Folic acid and vitamin B12 are essential vitamins for multiple functions in the body, and accumulating evidence suggests their importance in maintaining a high degree of methylation of the HPV genome, thus decreasing the likelihood of causing malignant lesions. HA, due to its re-epithelizing property, may prevent HPV virus entry in damaged mucosa and epithelia. Thereby, based on these premises, the combination of EGCG, folic acid, vitamin B12, and HA may be a very promising therapeutic approach to prevent HPV persistence.

## 1. Introduction

Epidemiological studies over the last decade have reported that human papillomavirus (HPV) infections are commonly sexually transmitted worldwide [1]. Recent data indicate that sexually active individuals will acquire an HPV infection at least once during their lives [2].

The pathogenic agents of HPV infection are papillomaviruses, non-enveloped circular double-stranded DNA viruses with a tropism for squamous epithelium and mucosal tissues [3]. Over 100 HPV types have been described, and 40 of them may infect the genital tract [4]. HPV infections may often cause cancer, mainly occurring at the transformation zones—“weak points”—between different types of epithelia [3]. HPVs are indeed the primary etiological agent for several cancers, including most of cervical cancers and anal, penile, vulvar, vaginal, and oropharyngeal tumors [5]. However, although HPV infection concerns both women and men, the high susceptibility to transformation following the infection, makes the weak point of the uterine cervix an ideal tissue to complete the viral lifecycle [6].

According to the oncogenic potential, papillomaviruses are classified into low-risk (LR) and high-risk (HR) HPV types. LR-HPV types, including types 1, 2, 6, and 11, are non-carcinogenic types as they do not induce cancerous lesions. They can lead to benign lesions (warts, condylomas, or recurrent respiratory papillomatosis) or, in rare cases, precancerous lesions [7]. Instead, HR types, including 16, 18, 31, 33, 35,45, 51, 52, 56, 58, 59, 66, and 68, are classified as carcinogenic types according to the IARC (International Agency for Research on Cancer) because they induce 99.7% of cervical cancers [8] and other anogenital cancers [9,10,11].

Often, HPV infections affecting cervical tissue can induce cervical intraepithelial (CIN) lesions of various degrees. These lesions, depending on their severity grade, can spontaneously revert or progress toward tumor development. Pre-malignant changes represent histological abnormalities ranging from atypical squamous cells of undetermined significance (ASCUS) and low-grade cervical dysplasia (LSIL/CIN1) that could progress to moderate dysplasia (CIN2) or severe dysplasia/carcinoma in situ (CIN3/CIS).

Following histologic diagnosis, the treatment of the HPV-induced lesions depends on the degree of severity: while ASCUS/CIN1 need no treatments at the time of diagnosis as most of them regress spontaneously, high-grade lesions (HSIL/CIN2 or CIN3) may undergo conservative surgical procedures, including ablative methods that destroy the affected cervical tissue [12]. Nevertheless, women with HSIL (CIN2/3) still have an increased risk of developing recurrent CIN2 or worse (CIN2+) after a loop electrosurgical excision procedure (LEEP) [13].

More than 90% of HPV infections are asymptomatic, transient, and regress within 6–12 months from the onset, thanks to the viral clearance activity of the immune system [14]. However, in some cases, the infection can persist over a year, thus increasing the probability of developing malignant tumors [14]. Prospective studies revealed that the prevalence of HPV includes a mix of both incident and persistent infections that have accumulated over time due to the lack of clearance [15].

Persistence is a peculiar concept in HPV infection, even though it is not homogeneously measured. Most authors refer to persistence as those infections having two consecutive positive HPV DNA tests with undetermined time intervals [14]; many other authors define persistence using the time to clearance, while others mean persistence as the same virus strain present in the same patient for more than nine months. Moreover, defining HPV persistence is further complicated considering the different methods of HPV laboratory detection, testing intervals, and HPV categorization [16]. However, despite the several definitions, persistence remains a key event in HPV infection, as it can trigger the development of malignant tumors. Persistence is associated with almost all cervical and anogenital cancers but also with head and neck cancers [17]. Several risk factors, such as genetics and lifestyle, can significantly increase the probability of developing persistent infections [18,19]. For instance, the carcinogens in cigarette smoke may increase the viral load and the probability of tumoral transformation in HPV-infected epithelial cells [20]. In addition, smoking or alcohol use are risk factors for persistent oral and genital HPV infections [21]. Given the prevalence of co-infections with multiple HPV types, several authors also indicated such factors as a predictor of persistent infection [22].

In general, the persistence of HPV infection—due to HR types—especially HPV types 16 (HPV16) and 18 (HPV 18)—is responsible for many genital [23] as well as oropharyngeal cancers [24]. If undetected and untreated, these types of lesions slowly progress in an average of 5–14 years due to the increased possibility of viral genome integration.

When HPV infection persists, genomic instability can increase viral genome integration into the host genome [25]. Once the viral genome has integrated into the host genome, this leads to a breakpoint in the E2 genetic sequence, resulting in the de-repression of the E6 and E7 viral oncogenes. The carcinogenic process, started with the E6 and E7 de-repression, includes the accumulation of additional alterations in the host genome that lead to the invasive cancer phenotype. For instance, E6 and E7 proteins impact the function of p53 and pRb proteins, two fundamental tumor suppressors [26,27].

Viral persistence often correlates with the ability to escape the immune response of the host. Indeed, the innate host immune response against HPV infection represents the first line of defense due to the recognition by pattern recognition receptors (PRRs) that activate downstream signaling pathways [28]. However, the HPV virus has developed evasion strategies to bypass immune surveillance, including the modulation of cytokine and chemokine expression, the alteration of antigen presentation process, and the down-modulation of the interferon (IFN) pathway and adhesion molecules [29]. Although integrated viral DNA is a hallmark of many HPV-associated cancers, in some cases, cancer cells may exhibit either integrated HPV DNA, extrachromosomal viral DNA, or a mix of both [30]. For instance, in HPV18-induced cancers, integrated sequences are prevalent, while in the case of HPV16-induced tumors, both genomic integration and the presence of episomes coexist [31].

To date, HPV persistence remains untargeted. The prevention of cervical cancer relies primarily on HPV vaccination (primary prevention) and other strategies (secondary prevention), such as cervical cancer screening programs, with the aim to detect and treat pre-cancerous lesions before they progress to cancer. Unfortunately, these strategies are not equally distributed worldwide [32], thus leaving millions of women without options to receive protection against HPV infections. Moreover, prophylactic HPV vaccines may reduce—but not eliminate—the risk of cervical cancer without altering the course of existing HPV infections.

Despite several advances in research and screening programs, HPV remains a global burden and health hazard since no specific existing therapies eradicate the virus nor counteract or prevent its persistence, which is considered the evolutionary success of the virus. Indeed, current treatments may only target clinical signs of the infection, such as condylomas or cervical lesions, while persistence still represents a therapeutic gap. From this perspective, developing new therapeutical strategies to counteract HPV infection and its persistence is a medical challenge. Therefore, this review aims to summarize all the current available scientific evidence about the role of epigallocatechin gallate (EGCG), folic acid, vitamin B12, and hyaluronic acid (HA) as a potential synergic treatment to prevent HPV persistence.

## 2. Methods

This review was reported and qualitatively assessed following the Scale for the Assessment of Narrative Review Articles (SANRA) [33].

The following databases were used to perform a non-systematic search: MEDLINE, EMBASE, Global Health, the Cochrane Library (Cochrane Database of Systematic Reviews, Cochrane Central Register of Controlled Trials, Cochrane Methodology Register), Health Technology Assessment Database and Web of Science, and research registers (such as www.clinicaltrials.gov). The following terms, “epigallocatechin gallate”, “folic acid”, “vitamin B12”, and “hyaluronic acid”, were each combined with “Human Papilloma Virus”. Only papers published in English, without any restrictions about the year of publication, were selected.

Two review authors (S.P. and E.L.) screened titles and/or abstracts of retrieved studies to identify the most pertinent ones for this narrative review. Two other team members (A.S.L. and A.F.) retrieved the full text of these articles and independently assessed them for eligibility. Disagreements between reviewers over the eligibility of a particular article were resolved through discussion with a third (external) collaborator. Two authors (S.G. and J.C.) independently extracted data from articles about study characteristics and included populations, types of intervention, and outcomes, using a pre-piloted standard form to ensure consistency. Any discrepancies were identified and resolved through discussion (with a third external collaborator where necessary). Due to the nature of the findings, we opted for a narrative synthesis of the results from selected articles.

## 3. Epigallocatechin Gallate

EGCG is one of the major bioactive polyphenolic components of green tea—known as catechins—with immunostimulatory, antioxidant, antiproliferative, and pro-apoptotic activity [34]. In the context of HPV infections, extensive research, both in vitro and in vivo, reported the role of EGCG in regulating viral infection and preventing cervical cancer.

Several in vitro studies demonstrated that EGCG has an antiproliferative activity by interfering with the HPV life cycle and suppressing the oncogenes and oncoproteins E6/E7, which are responsible for the viral oncogenic activity and cancer development. In particular, a recent in vitro study reported that EGCG inhibits the growth of premalignant HPV18-positive keratinocytes by stimulating the degradation of E6 and E7 proteins through the ubiquitin-proteasome pathway [35]. The suppression of E6 and E7 proteins correlates with the up-regulation of tumor-suppressor genes as p53, pRb, and p21 [36,37,38], thus resulting in the apoptosis of the cervical cancer-derived cell line, Caski cells (HPV16 positive) and Hela cells (HPV18 positive). Indeed, both E6 and E7 interfere with cell cycle regulation by binding p53 and pRB, thus preventing apoptosis [39]. Interestingly, beyond the modulation of p53 and pRb in Caski cells (HPV16 infected) and Hela cells (HPV18 infected), EGCG may target most of the mechanisms involved in cancer transformation and progression, such as cellular proliferation, microtubule stability, angiogenetic processes, and cellular apoptosis. According to a recent work, a dosage of 100 µM of EGCG inhibits telomerase activity—as an effective method for anticancer protection—in different cellular models of HPV infection: (i) HPV18-immortalized ectocervical cells (HEC-18), (ii) transformed HPV18-immortalized human ectocervical cells (HEC-18T), (iii) HPV18-immortalized endocervical cells (HEN-18), and (iv) serum-adapted HPV18-immortalized human endocervical cells (HEN-18S). These results highlighted the ability of EGCG to reduce the cellular growth rate in human HPV-infected endocervical and ectocervical cells [40]. Furthermore, increasing concentrations of EGCG (10 uM, 25 uM, and 50 uM) for 24, 48, and 72 h inhibited cell proliferation and modulated RNA polymerase III in a dose-dependent manner in HeLa cells (HPV18-infected) [41]. Since microtubule cytoskeletal structures play a key role in proliferation, signalling, and migration in cancer cells [42], microtubules and tubulin—as their monomer—are popular targets for anticancer drugs [43]. In this regard, microtubule depolymerization is another mechanism targeted by the antiproliferative activity of EGCG in HeLa cells (HPV18-infected) [42]. Indeed, the IC50 dose (50 mM) rapidly disrupted microtubule networks, and higher doses (75 and 100 mM) of EGCG drastically distorted microtubule structure, making the cells almost round-shaped. In addition, other studies on HeLa cells revealed that EGCG treatment prevents the spreading of cancer cells by keeping their round shape and reducing in a dose-dependent manner the expression of metalloproteinases (MMP-2 and MMP-9). These latter, by degrading various components of the extracellular matrix (ECM), play a critical role in cancer invasion, migration, metastasis, and tumorigenesis [44,45]. EGCG also suppresses the angiogenetic process through which tumoral cells obtain nutrients and oxygen by down-modulating the expression of vascular endothelial growth factor (VEGF) [46].

In addition, EGCG can prevent cancer progression by inducing apoptosis in cervical cancer cells. In vitro studies in both HeLa and SiHa cells (HPV16 positive) demonstrated that EGCG promoted apoptosis in a time-dependent manner by increasing the expression of pro-apoptotic genes such as p53 and caspase-3 [47,48]. Another mechanism by which EGCG stimulates apoptosis in cancer cells is through mitochondrial perturbation, correlated to an excess in hydrogen peroxide. A dosage of 60 mM EGCG for 12 h in serum-free medium HeLa cells can trigger the permeabilization of the lysosomal membrane. Hence, lysosomal proteases and hydrolytic enzymes are released into the cytosol leading to HeLa cell death [49].

A recent paper highlighted the activity of EGCG to counteract one of the escaping mechanisms of HPV. Indeed, the virus can escape the immune system surveillance by interrupting the type I IFN signalling pathway and establishing a persistent infection. When keratinocytes are transfected with type 2 HPV (HPV2) E7, the mRNA and protein expression of type I IFN signaling pathway components are significantly downregulated. EGCG pre-treatment can reverse it by significantly up-regulating them, so reinforcing innate antiviral immunity against HPV2 [50].

Despite this molecular evidence, different clinical studies demonstrated the efficacy of EGCG, both as a topical and oral formulation, in recovering HPV infections. Tatti and colleagues reported that an EGCG-based formulation is effective for topically treating external genital warts, which are a cutaneous manifestation of a proliferative disorder due to LR subtypes, such as HPV6 and HPV11 [51]. Two clinical trials at phase III organized as multicenter, randomized, double-blind, and controlled, evaluated the effectiveness of treatment with EGCG-based ointment (10% or 15%) on external genital warts showing a reduction in baseline warts area by at least half, compared with 52.2% in the control group (*p* < 0.05) [51].

Later, a systematic review and meta-analysis [52], including three randomized studies, evaluated the positive effects of an EGCG-based topical formulation in the treatment of warts. These studies screened a total of 660 men and 587 women and confirmed the efficacy and safety of EGCG for the topical treatment of genital warts [53,54,55].

Besides these beneficial effects on external warts, another randomized controlled-clinical study investigated the efficacy of green tea extracts in different forms in patients with HPV and pre-cancerous lesions of various degrees. Ahn and collaborators demonstrated that after 8–12 weeks of oral intake of EGCG (200 mg), cervical lesions regressed, and the clearance of the virus increased, thus preventing the progression toward a more severe grade [56]. A total of 6 among 10 patients receiving EGCG capsules orally exhibited a positive response, in contrast to the untreated control group, in which only 4 of the 39 patients exhibited a positive response. As stated by the authors, about 10% of the response rate in the untreated group may correlate to differences in the immune status of the patients or other unknown factors.

Taken together, these data emphasize the powerful antiviral activity of EGCG in individuals with HPV infection. Overall, EGCG mediates antiproliferative, antioxidant, antimetastatic, and pro-apoptotic activities in HPV-infected cell lines, leading to beneficial effects in HPV-infected patients and representing a promising molecule in the treatment of HPV persistence.

## 4. Folic Acid and Vitamin B12

Folic acid and vitamin B12, also respectively known as vitamin B9 and cobalamin, are water-soluble vitamins that are not synthesized by the human body. Instead such vitamins are derived from dietary sources such as meat, fish, and dairy products, as well as cereals. Poor dietary intake of folic acid and vitamin B12 can be a cofactor for HPV-induced cancers [57,58,59,60,61] due to their crucial role in DNA synthesis and repair.

Human folate deficiency correlates with several health disorders, including neural tube defects, vascular disease, microcytic anemia, and many types of cancer, including oral cancers. Moreover, insufficient dietary intake of folic acid may lead to dysregulation of DNA methylation, thus interfering with DNA synthesis and repair, which may trigger these adverse health conditions [62]. Both folic acid and vitamin B12 inversely correlate with homocysteine levels and increased risk of cervical cancer. Lower serum folate and higher homocysteine levels might increase the susceptibility to cervical cancer [59], as homocysteine levels correlate with the severity of cervical dysplasia and HPV infection persistence. Indeed, women with HSILs (HPV+) and LSILs (HPV+) exhibit higher serum levels of homocysteine than the control group, and the more the cervical dysplasia increases, the more the serum homocysteine levels increase, especially in the presence of HPV [63]. Moreover, the blood serum of HPV-positive patients with CIN3 had significantly lower levels of folic acid and higher levels of free homocysteine [64].

Folic acid influences gene stability and expression by promoting DNA methylation [65], an epigenetic regulatory pathway that modulates viral gene expression and determines the fate of virus-infected cells. Demethylated viral DNA characterizes exclusively tumorigenic cells, while hypermethylated DNA is typical of non-malignant tissues [66], suggesting demethylation as a hallmark of cellular transformation [67].

Both folic acid and vitamin B12 are necessary for the synthesis of S-adenosylmethionine (SAM), which acts in various methylation reactions as the main donor of methyl groups [68]. Low levels of folic acid decrease DNA methylation and consequently increase the frequency of fragile sites on DNA [69]. Indeed, both folate and vitamin B12, by maintaining genomic stability, may prevent the integration of HPV into cervical cells’ genomes. This is an early event in HPV-linked carcinogenesis and a crucial step towards malignant progression, contributing to impairing the existing balance in host–virus interaction during latency [70].

DNA hypomethylation of cervical tissue also correlates with CIN severity. Low-grade dysplasia exhibits a higher degree of DNA methylation than high-grade lesions and cancer, which correlates with significant DNA hypomethylation. Indeed, women with CIN-HPV have statistically lower levels of folic acid, which correlates with more severe lesions [71]. These data evidence how global hypomethylation is an early epigenic event in cervical carcinogenesis. Subsequent research confirmed this aspect demonstrating that in vitro methylation selectively down-regulates the transcription of HPV18 genome [72].

Folic acid and vitamin B12 deficit predisposes genome fragmentation [73] and induces breaks within fragile sites, which are the same fragile sites preferential for HPV16 integration [74]. Animal models confirm that dietary folate deficiency can profoundly influence and modulate events leading to HPV16-induced carcinogenesis and facilitate genomic integration of HPV16 DNA [75].

Clinical studies demonstrated that, in the case of folate deficiency, HPV persistent infection and progression of cervical dysplasia increase [76]. HR-HPV-positive women usually exhibit high plasma concentrations of folate or vitamin B12 and rarely undergo a diagnosis of CIN2 [77]. Serum levels of vitamin B12 and folate in ASCUS (+) HPV (+) patients are significantly lower than in ASCUS (+) HPV (−) patients [78]. Moreover, higher levels of folate and vitamin B12 are inversely correlated with a positive diagnosis of HR-HPV. Subjects with higher folate and vitamin B12 status are 73% less likely to become test-positive for HR-HPV types and more likely to become test negative [61,76].

Hence, considering that HPV infection correlates with low serum levels of vitamin B12 and folate, monitoring their levels in patients with cervical lesions may help to identify a persistent infection. On these premises, supplementing vitamin B12 and folic acid could be a valid strategy to counteract HPV genome integration and, consequently, its persistence.

## 5. Hyaluronic Acid

HA is a major component of the extracellular matrix (ECM) in epithelial and connective tissues. HA is a very peculiar molecule: despite its simple chemical structure, it has several functions depending on its molecular weight. Indeed, HA can be synthesized in high molecular weight (HMWHA) (>500 kDa) and in low or very low molecular weight (LMWHA or vLMWHA <500 kDa, <10 kDa, respectively).

In homeostatic conditions, HA is mainly synthesized as HMWHA, and it has important biological properties and functions. It is a tissue-hydrating lubricant [79] with anti-angiogenic [80] and anti-inflammatory properties [81]. Moreover, HMWHA exhibits immunosuppressive activities since it creates a dense and viscous protective “coat” that covers the surface of cellular receptors, such as the Toll-like receptors (TLRs), thus preventing the activation of the inflammatory reaction.

HPV infection is mainly acquired through sexual intercourse. Moreover, the virus spreads during sexual interaction through close skin-to-skin contact, which preferably involves areas that receive trauma and/or minor injuries. Indeed, the loss of continuity in the epithelia allows the viral particles to penetrate and infect [82]. Therefore, preserving or restoring the integrity of cervical tissue may represent one of the goals for preventing HPV infections. Specifically, LMWHA and vLMWHA may contribute to reaching this target. For instance, both of them find application in the gynecological field to maintain vaginal function and women’s well-being [83]. When locally applied, epithelia may absorb LMWHA easier than HMWHA [84], and when orally taken, it can significantly increase vaginal epithelial thickness in postmenopausal women [85]. In fact, both LMWHA and vLMWHA promote the process of wound-healing repair by stimulating the production of pro-inflammatory factors [86,87]. In this way, they decrease the contracture in wound scars [88] and favor collagen expression [89]. This is a therapeutic strategy for endometrium regeneration in cases of endometrial trauma [90]. Indeed, as reported in a recent clinical study, HA accelerates the re-epithelization process in human skin wounds compared to pre-treatments with saline solution [91]. Moreover, in the case of florid oral papillomatosis, the treatment with HA gel helps to regenerate the perilesional mucosa, thus preventing the occurrence of side effects and reducing the need for additional surgical intervention, with the consequent improvement in patients’ quality of life [92].

To date, the application of vaginal HA represents one of the most used approaches to restore the re-epithelization of the cervix, thus helping spontaneous viral clearance, mainly in conditions of LSILs. For the first time, a recent randomized controlled trial provided evidence for using HA in association with other compounds to boost their efficacy. Indeed, the association of vaginal HA-based soft gel capsules with orally taken Echinacea extracts reduced the number of remaining CIN1 samples and improved histological, cytologic, and colposcopic outcomes with respect to vaginal HA or *Echinacea purpurea* (EP) plus *Echinacea angustifolia* (EA) alone [93]. Only combining the oral supplementation of Echinacea with the administration of the HA soft gel caps seemed to boost viral clearance and reduce the persistence of LSIL/CIN1 lesions during the 12-month follow-up. This evidence confirms that HA not only may act as a preventive approach to inhibit virus infection, but it also represents a pivotal adjuvant approach to boost the efficacy of other compounds and decrease the number of lesions [93].

## 6. Conclusions

To date, HPV infection still represents a global burden. No medical approach effectively eradicate the virus, instead the available medical strategies only prevent or treat restricted clinical manifestations without targeting persistence. This latter—persistence—is the most important risk factor of tumor development, as in the case of cervical cancer, and it still represents a serious clinical challenge. Accumulating evidence suggests that some natural molecules may be beneficial in treating HPV-induced lesions, thus opening the possibility to consider them as a potential therapeutic strategy to also prevent HPV persistence.

**Figure 1:**
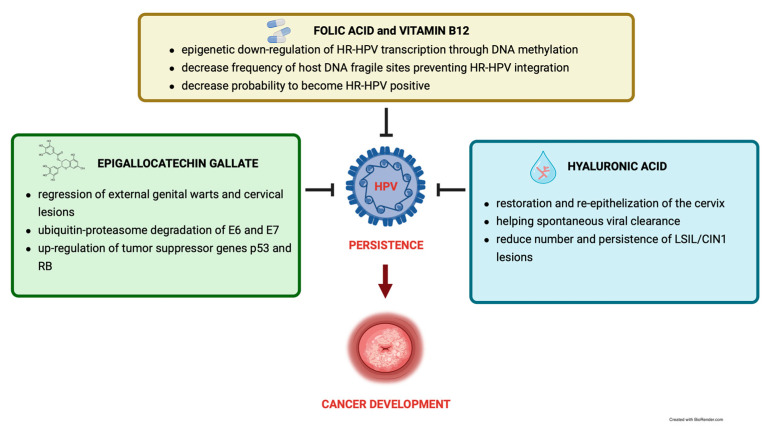
The synergic effect of epigallocatechin gallate (EGCG), folic acid (FA), vitamin B12 (B12), and hyaluronic acid (HA) in preventing HPV persistence.

The identified natural molecules—EGCG, vitamin B12, folic acid, and HA—rely on several scientific pieces of evidence demonstrating the antiviral action of each molecule in the context of HPV infections. These results indicate that each of the molecules display key properties in counteracting HPV infection, thus suggesting their combination as a valid therapeutic approach to prevent and counteract HPV persistence. The combination of the antiproliferative effect of EGCG in cervical lesions, or the induced-up regulation of p53 and Rb genes (usually de-regulated in a persistent HPV infection), the potential of vitamin B12 and folic acid to prevent the viral genome integration (a peculiar characteristic of HR-HPV persistence) and the re-epithelizing properties of HA or its boosting activity, suggest a promising therapeutical approach against HPV infection and most of all, against its persistence (Figure 1).

Nevertheless, no clinical studies still investigated their association. Therefore, new clinical evidence results are necessary to deepen the promising role of these combined natural molecules against HPV persistence.

## Data Availability

Not applicable (no data was generated during the writing of this review).
